# Retraction: Among live and dead bacteria, the optimization of sample collection and processing remains essential in recovering gut microbiota components

**DOI:** 10.3389/fmicb.2025.1654634

**Published:** 2025-07-07

**Authors:** Frontiers Editorial Office

The journal retracts the July 10 2019 article cited above.

Following publication, it was brought to our attention that the ethical approval for this study referred to the same ethics approval number 2016-011 that had been used across a series of unrelated published studies (Afouda et al., 2020, 2019; Amrane et al., 2018, 2019; Anani et al., 2019; Andrieu et al., 2018; Bellali et al., 2020, 2021; Belkacemi et al., 2020, 2018; Benabdelkader et al., 2019; Diakite et al., 2019, 2020; Dione et al., 2018; Dubourg et al., 2020; Fall et al., 2019, 2018; Francis et al., 2019; Grine et al., 2019, 2018; Guindo et al., 2020; Hamad et al., 2018, 2017; Hassani et al., 2020; Hocquart et al., 2019; Kuete Yimagou et al., 2019; Kuete et al., 2019a,b; Maaloum et al., 2022b,a; Mbogning Fonkou et al., 2019; Mekhalif et al., 2019; Naud et al., 2021; Ndongo et al., 2022; Ngom et al., 2020; Senghor et al., 2018, 2019, 2017b,a; Takakura et al., 2020, 2019; Traore et al., 2019; Yimagou et al., 2019b, 2020, 2019a). It remains unclear whether appropriate ethics provisions, in line with Frontiers Policies and Publication Ethics policies were adhered to.

The authors and a representative of Aix-Marseille Université Ethics Committee were not able to address all our concerns. As such, this article is retracted.

This retraction was approved by the Chief Executive Editor of Frontiers.

The authors do not agree with this retraction.
